# Deficient Plakophilin-1 Expression Due to a Mutation in *PKP1* Causes Ectodermal Dysplasia-Skin Fragility Syndrome in Chesapeake Bay Retriever Dogs

**DOI:** 10.1371/journal.pone.0032072

**Published:** 2012-02-22

**Authors:** Thierry Olivry, Keith E. Linder, Ping Wang, Petra Bizikova, Joseph A. Bernstein, Stanley M. Dunston, Judy S. Paps, Margret L. Casal

**Affiliations:** 1 Department of Clinical Sciences, College of Veterinary Medicine, North Carolina State University, Raleigh, North Carolina, United States of America; 2 Center for Comparative Medicine and Translational Research, College of Veterinary Medicine, North Carolina State University, Raleigh, North Carolina, United States of America; 3 Department of Population Health and Pathobiology, College of Veterinary Medicine, North Carolina State University, Raleigh, North Carolina, United States of America; 4 Section of Medical Genetics, School of Veterinary Medicine, University of Pennsylvania, Philadelphia, Pennsylvania, United States of America; 5 Long Green Animal Dermatology Center, Baldwin, Maryland, United States of America; University Hospital Hamburg-Eppendorf, Germany

## Abstract

In humans, congenital and hereditary skin diseases associated with epidermal cell-cell separation (acantholysis) are very rare, and spontaneous animal models of these diseases are exceptional. Our objectives are to report a novel congenital acantholytic dermatosis that developed in Chesapeake Bay retriever dogs. Nine affected puppies in four different litters were born to eight closely related clinically normal dogs. The disease transmission was consistent with an autosomal recessive mode of inheritance. Clinical signs occurred immediately after birth with superficial epidermal layers sloughing upon pressure. At three month of age, dogs exhibited recurrent superficial skin sloughing and erosions at areas of friction and mucocutaneous junctions; their coat was also finer than normal and there were patches of partial hair loss. At birth, histopathology revealed severe suprabasal acantholysis, which became less severe with ageing. Electron microscopy demonstrated a reduced number of partially formed desmosomes with detached and aggregated keratin intermediate filaments. Immunostaining for desmosomal adhesion molecules revealed a complete lack of staining for plakophilin-1 and anomalies in the distribution of desmoplakin and keratins 10 and 14. Sequencing revealed a homozygous splice donor site mutation within the first intron of *PKP1* resulting in a premature stop codon, thereby explaining the inability to detect plakophilin-1 in the skin. Altogether, the clinical and pathological findings, along with the *PKP1* mutation, were consistent with the diagnosis of ectodermal dysplasia-skin fragility syndrome with plakophilin-1 deficiency. This is the first occurrence of ectodermal dysplasia-skin fragility syndrome in an animal species. Controlled mating of carrier dogs would yield puppies that could, in theory, be tested for gene therapy of this rare but severe skin disease of children.

## Introduction

Congenital and hereditary disorders of skin adhesion are collectively grouped under the umbrella of epidermolysis bullosa (EB), and these diseases are further separated depending upon the level of intracutaneous cleavage [1]. A recent consensus classification distinguished four major types of human EB, which included EB simplex (EBS), junctional EB, dystrophic EB and the Kindler syndrome; these variants exhibit skin clefting respectively in the epidermis itself, the basement membrane's lamina lucida, the sub-lamina densa or multiple areas [1]. Among the subtypes of EB simplex, the suprabasal variants now include lethal acantholytic EB due to mutations of the gene encoding desmoplakin, the ectodermal dysplasia-skin fragility syndrome (ED-SFS) with plakophilin-1 (PKP1) deficiency and EBS superficialis of unknown genetic cause [1].

First described in 1997 by McGrath et al. [2], ED-SFS (OMIM #604536) is a genodermatosis that has, to our knowledge, been reported in only 12 human patients [2]–[10]. Skin lesions were first noticed at birth in all but one individual [5]. Affected neonates initially exhibit diffuse erythroderma and skin fragility (blisters, erosions) at areas of friction and trauma [11]. Chronic perioral inflammation (cheilitis) with cracking and palmoplantar keratodermas with fissures are often seen; all cases have been reported to also have hair abnormalities such as partial hypotrichosis to complete hairlessness, woolly hair as well as nail dystrophies [11]. Children with ED-SFS are generally of short stature, but there is no recognized associated cardiac pathology as in several other desmosomal genodermatoses [11]. Microscopic lesions include epidermal hyperplasia with cell-cell separation that varies from a widening of intercellular spaces to severe acantholysis in the stratum spinosum. Aggregation of keratin filaments (e.g. dyskeratosis) is also seen [12]. Ultrastructurally, the desmosomes are small and keratin filaments appear to separate from the desmosomal inner plaques [12]. In these patients, the expression of PKP1 is reduced to absent and that of desmoplakin changes from a typical intercellular to a cytoplasmic pattern. Several mutations have now been reported (compiled in [11]), with nearly all of them leading to a complete loss of expression of PKP1. Individuals with one normal allele appear phenotypically normal [7].

Spontaneously arising hereditary skin diseases resembling suprabasal variants of human EBS have been reported only exceptionally in animals. A disease similar to human acantholytic EB has so far been described only in cattle: the so-called “familial acantholysis” was first reported in Angus calves nearly 30 years ago [13], while “hereditary suprabasal acantholytic mechanobullous dermatosis” was seen more recently in Brazilian Murrah buffaloes [14]. A third occurrence of a similar phenotype was described briefly in Texas Brangus calves (cited in [15]). In none of these examples, however, has the genetic cause of acantholytic EB been determined. At this time, a spontaneous or experimental animal model of human ED-SFS with PKP1 deficiency has not been reported.

In this paper, we characterize a novel genodermatosis of juvenile Chesapeake Bay retriever (CBR) dogs that clinically, histologically and ultrastructurally reproduces the phenotype of human ED-SFS. In these dogs, a mutation of the canine orthologue of *PKP1* was found to cause the absence of PKP1.

## Materials and Methods

### Ethics Statement

Researchers were not at all involved in the breeding of these dogs. Outside of a standard physical examination, the authors did not perform any research-related work before death. Due to the pain and incurability of their disease, the puppies were euthanized within a couple of hours of their arrival to our hospital.

### Histopathology

Skin samples from neonatal CBR puppies, and skin and internal organ samples collected at post mortem examination from three month-old CBR puppies, were fixed in 10% neutral-buffered formalin, processed routinely in paraffin, cut to 5 micrometer histological sections and stained with hematoxylin and eosin for histopathology evaluation. Per NC State University's Animal Care and Use Policy, an application for vertebral animal use was not required as tissues were collected after the animals' death.

### Electron Microscopy

For transmission electron microscopy (TEM), 1 mm thick skin samples from carpal pad, trunk and concave aspect of ear pinnae of three month-old CBR puppies (a total of three dogs) were placed in McDowell's and Trump's fixative (four parts formaldehyde to one part glutaraldehyde) within less than 30 min of euthanasia. Samples were rinsed twice with 0.1 M sodium phosphate buffer (pH 7.2), prior to incubation in 1% osmium tetroxide (EMS 19110, Hatfield, PA) in 0.1 M phosphate buffer for one hour, rinsed twice with distilled water, and dehydrated in a concentration gradient series of ethanol baths, followed by 100% acetone double rinse. Samples were then placed in a mixture of Spurr resin (EMS Spurr resin kit 14300, Hatfield, PA) and acetone (1∶1) for 30 minutes, followed by two changes of 100% resin for two hours each. Subsequently, samples were polymerized in resin at 70°C for eight hours. Semi-thin sections (0.25 µm) were cut, stained with 1% toluidine blue-O in 1% sodium borate and used to confirm epidermal areas. Ultrathin sections (70–90 nm) were stained with methanolic uranyl acetate (EMS 22400, Hatfield, PA), followed by lead citrate, and were examined by transmission electron microscope (Philips EM208S TEM, Oregon, USA). Reagents were obtained from Fisher Scientific, Pittsburgh, PA, unless otherwise indicated.

### Protein Immunomapping

The presence and expression pattern of selected keratin and desmosomal adhesion molecules was assessed on biopsies from clinically affected and unaffected footpad and ear skin. We performed indirect IF using a panel of antibodies specific for the human proteins. Details on the validation of these antibodies for use in dogs can be found in a recent article [16]. Additional antibodies were also used: the mouse antihuman K10 monoclonal antibody DE-K10, known to recognize the canine homologue, was from Santa Cruz Biotechnology (#sc-52318; Santa Cruz, California); it was used at 1∶5 dilution overnight at room temperature. Mouse antihuman K14 monoclonal antibody clone LL002 (#AM146-5M, BioGenex, San Ramon, California), used undiluted for 2 hrs at room temperature, has been shown previously to recognize the canine protein [17]. Finally, to verify the absence of PKP1 staining in affected dogs, we repeated the immunostaining for this protein using a monoclonal antibody specific for the conserved armadillo repeats of PKP1 (PP1-5C2 undiluted for 2 hrs; Mybiosource, California). Fluorochrome-labelled secondary antibodies were used as described previously [16].

### Mutation Analysis

Tissue and blood samples were obtained from three CBRs with ED-SFS, two pairs of their parents, ten clinical normal littermates and related dogs, as well as one unrelated normal dog. Genomic DNA was extracted using the QIAamp DNA Extraction kit (QIAGEN, Valencia, CA) following manufacturer's protocol.

All 13 exons of *PKP1* spanning 4021 base pairs were sequenced using genomic DNA samples from an affected CBR, its sire, and an unrelated normal dog (Table S1). Standard polymerase chain reactions (PCRs) were performed to amplify exons 2–8a, 9, 12 and 13. Briefly, the reaction mixture contained 50 ng of genomic DNA as template, 2.5 µl of 10× PCR buffer, 0.75 µl of 50 mM MgCl_2_, 0.5 µl of 10 mM dNTPs (2.5 mM each), 0.5 µl of each 20 µM primers (forward and reverse), 0.25 µl of platinum Taq polymerase (5 U/µl) and sterile de-ionized water (diH_2_O) up to a total volume of 24 µl (all reagents were from Invitrogen, Carlsbad, CA except for the primers that were designed by one of the authors (PW) and synthesized by Integrated DNA Technologies, Inc., San Diego, CA). The general PCR conditions were as follows: initial denaturing at 94°C for 5 minutes, followed by 35 cycles of 94°C for 30 seconds for denaturing, 62°C for 30 seconds for annealing (Table S1), and 72°C for 45 seconds for extension. Amplification of exons 1, 8, 10 and 11 required alternate reagents because of the GC-rich sequences. For exon 1, a hot-start KOD PCR kit was used (Novagen, Madison, WI); for exon 8, 10 and 11, 5× buffer E with higher pH (Invitrogen) was used instead of regular 10× buffer. Polymerase chain reaction products were purified after 1% agarose gel electrophoresis using the QIAquick gel extraction kit (Qiagen, Valencia, CA), and sequenced at the Gene Sequencing Center of the Medical School of University of Pennsylvania. Sequencing data was analyzed using Lasergene software (DNAStar, Madison, WI).

Once an informative nucleotide polymorphism was discovered, endonuclease reactions were carried out using Alu1 restriction enzyme to distinguish the variants. The digestion reaction mixture included NEB Buffer 4 (10×), AluI (10,000 U/ml) and dIH_2_O at a ratio of 2∶0.25∶7.75 (New England Biolabs, Ipswich, MA). Of this mixture, 10 µl was mixed with 10 µl of the PCR product. Digestion took place in a 37°C water bath overnight; the digested products were separated on a 1% agarose gel by electrophoresis and stained with ethidium bromide for visualization.

## Results

### Pedigree Analysis

Between 2008 and 2009, nine (four males, five females) out of 28 CBR puppies were diagnosed with ED-SFS; six of the affected puppies died within hours or days of birth. The affected puppies were born in four different litters to eight related, clinically normal parents. A seven-generation pedigree was evaluated in which all of the affected dogs could be traced back to one common ancestor. The degree of inbreeding ranged from 0.031 to 0.125 (mean = 0.036), which is fairly typical of a tightly bred purebred dog population. One affected dog (C18) was produced from a mother-son mating (inbreeding coefficient = 0.25). Pedigree analysis was most consistent with an autosomal recessive mode of inheritance.

### Clinical Synopsis

**Figure 1:**
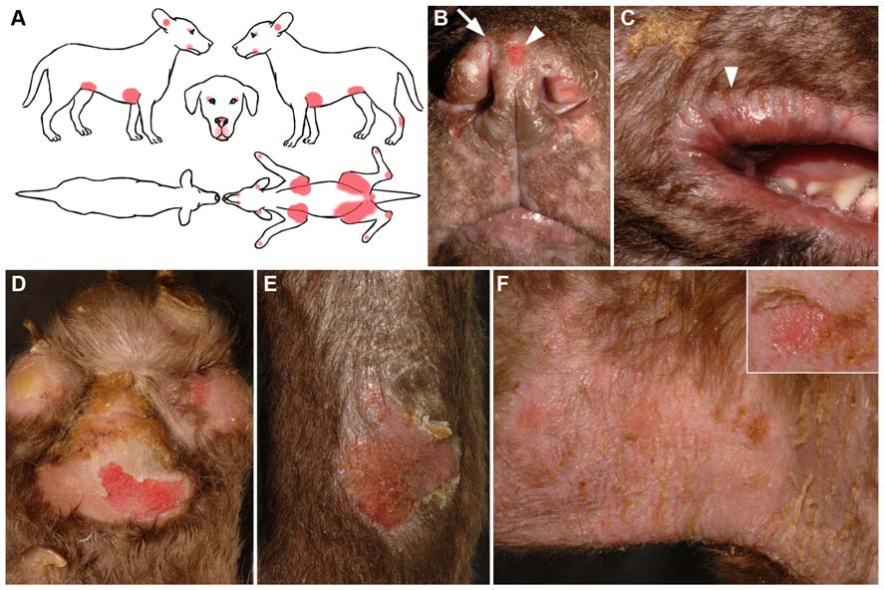
Clinical characteristics of dogs with ectodermal dysplasia-skin fragility syndrome with PKP1 deficiency. Panel A: silhouette with lesion distribution of a 3-month-old puppy: skin lesions (highlighted in red) are localized at mucocutaneous junctions of the face (periocular, nose, lips, ear openings) and areas of friction on the axillae, groin, joints and footpads. Panel B: erosions of the nasal planum (arrowhead) and dorsal nasal fissure (arrow). Panel C: fissures around the lips (cheilitis, arrowhead). Panel D: footpad erosions due to sloughing of the superficial epithelium. Panel E: erosions and crusts on the extensor aspect of the hock. Panel F: superficial erosions and crusts with vertical fissuring on the right axilla. Inset: rubbing of normal appearing skin with a pencil eraser led to erosion formation (positive direct Nikolskiy sign).

At birth, the skin of affected dogs was abnormally pale and translucent on the ears, feet, nose and mouth area, otherwise the puppies appeared normal. In all affected dogs, clinical signs occurred immediately after birth with spontaneous sloughing of the nose and footpad epithelium and bleeding of the ear tips if traumatized. Within 48 hours of birth, the lips and facial superficial skin layers also sloughed when rubbed dry or licked by the mother. Three dogs were kept alive by their breeder for three months; all exhibited waxing and waning superficial skin sloughing with erosions and fissures at areas of friction (axillae, groin, caudal tarsi, footpads), concave ear pinnae and mucocutaneous junctions (nasal planum, philtrum, lips, periocular area) (Figure 1). Rubbing normal-appearing skin with a pencil eraser elicited the formation of erosions (positive direct Nikolskiy sign). Footpads exhibited irregular hyperkeratosis and some claws appeared small and dystrophic. Footpad lesions resulted in occasional limping. At three months of age, two of these three dogs were approximately one third of the size and weight of their normal littermates. Their hair coat was finer and appeared abnormal compared to that of normal littermates; there were patches with partial absence of hair. The three 3-month-old puppies were euthanized for humane reasons because of poor quality of life. Complete post-mortem examinations revealed, in addition to the skin lesions described above, multifocal, oval to linear, oral erosions and ulcers that tracked the frictional margins of the tongue and superficial ridges of the hard palate. Subtle esophageal erosions were present. Lesions of other internal organs were not seen.

### Histopathology

**Figure 2:**
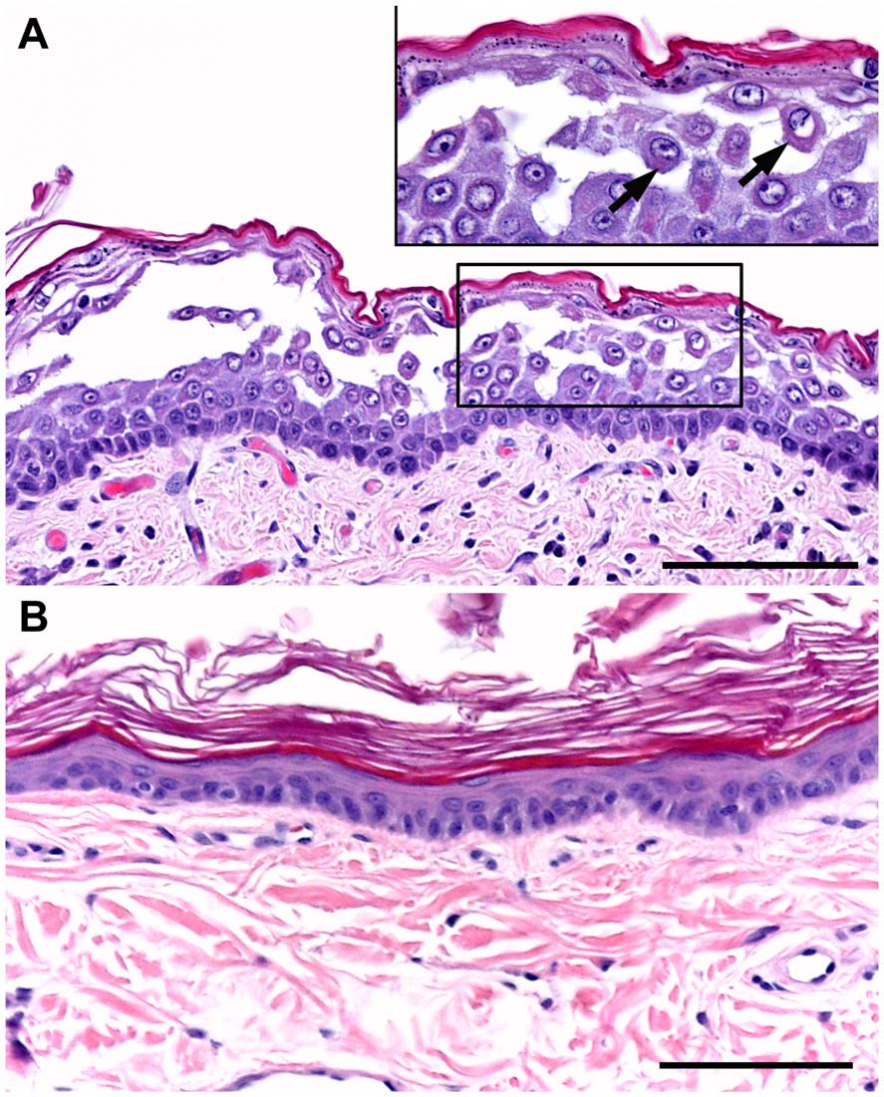
Histopathology findings for neonatal puppies with ectodermal dysplasia-skin fragility syndrome and PKP1 deficiency. Panel A: coalescing keratinocyte acantholysis disrupts epidermal architecture in a biopsy from the distal limb with prominent involvement of the stratum spinosum. At higher magnification (insert), individual acantholytic keratinocytes (arrows) are rounded and have retraction and condensation of eosinophilic tonofilaments within the cytoplasm. Hematoxylin and eosin. Panel B: in normal canine skin, the epidermis consists of keratinocytes abutting each other at all layers. Bar = 100 µm.

**Figure 3:**
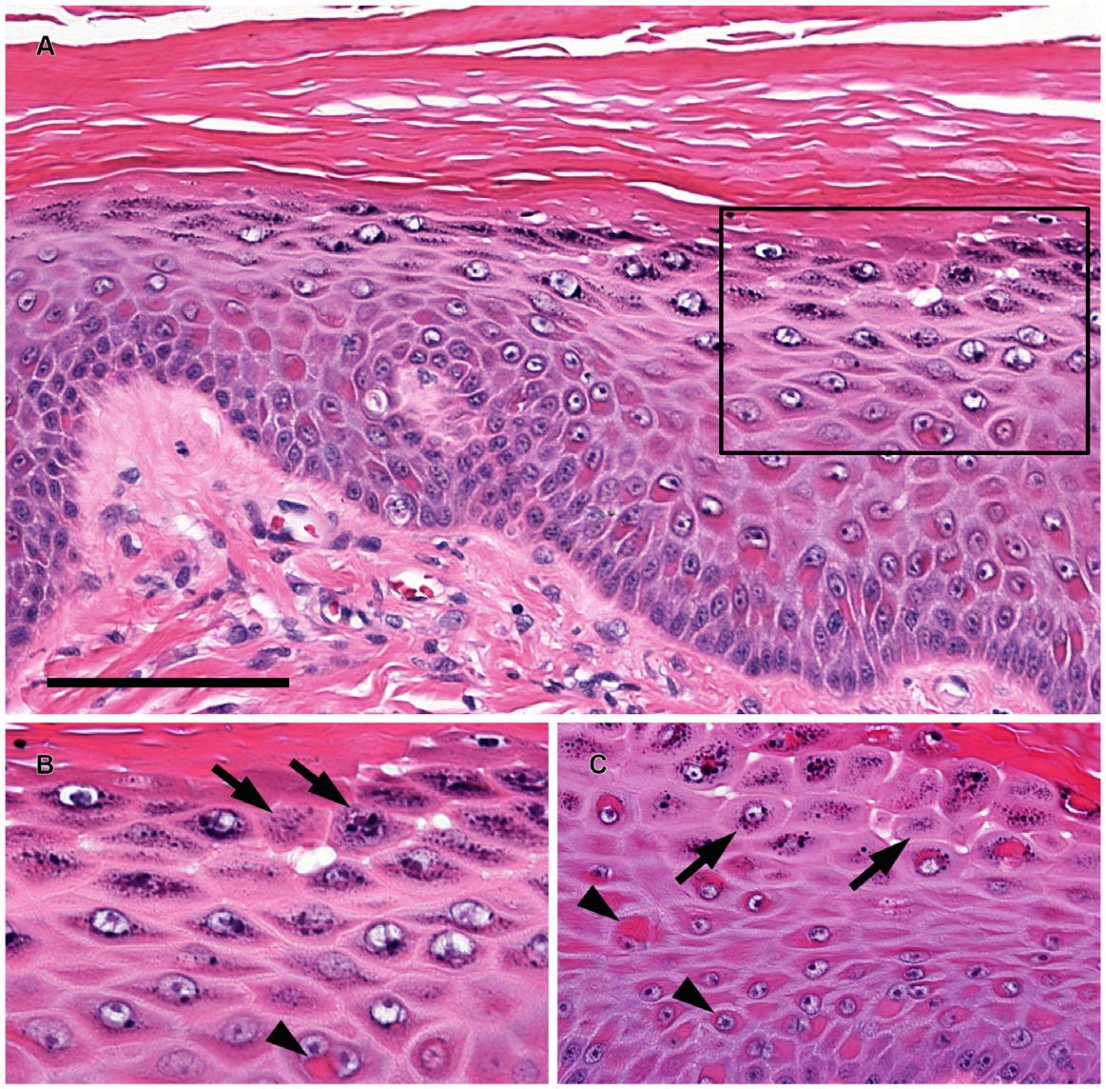
Histopathology findings for 3-month-old puppies with ectodermal dysplasia-skin fragility syndrome and PKP1 deficiency. Panel A: Trunk skin exhibits diffuse epidermal hyperplasia topped by prominent, laminated to compact, orthokeratotic hyperkeratosis. Panel B: Higher magnification photomicrograph (Box insert from Panel A) illustrates mild keratinocyte acantholysis (arrows) in the stratum granulosum as well as condensation and aggregation of eosinophilic tonofilaments in the cytoplasm (arrow head). Panel C: Similar changes of keratinocyte acantholysis (arrows) and condensation of tonofilaments (arrowheads) are observed in the carpal pad epidermis. Hematoxylin and eosin. Bar = 100 µm.

In neonates that died within hours of birth, histopathology of the ear, trunk, distal limb and footpad areas revealed separation of keratinocytes and common, multifocal acantholysis of suprabasal keratinocytes, which coalesced in some areas (Figure 2A). This dyscohesion is in marked contrast with the architecture of normal canine skin in which cell-cell contacts are undisturbed (Figure 2B). In three month-old puppies, the epidermis at multiple body sites, including from lesions on the trunk, limbs, face, and footpads, was diffusely hyperplastic and topped by prominent laminated to compact, orthokeratotic hyperkeratosis (Figure 3). Subtle separation of keratinocytes was present. Overt keratinocyte acantholysis was uncommon; it was present most often in the stratum granulosum where it was seen as mild rounding and early separation of keratinocytes (Figure 3). Neonates and 3-month-old puppies shared additional histological features. Acantholytic and nonseparated keratinocytes often contained perinuclear eosinophilic rings and rounded cytoplasmic aggregates (Figures 2, 3) supporting the hypothesis of tonofilament network collapse and aggregation; this was more dramatic in older puppies, especially in footpad epidermis (Figure 3). Peripheral cytoplasmic pallor accompanied this change. Inflammation was not a feature of early lesions; however, lesions that progressed to secondary deep erosions and ulcers in friction prone areas developed neutrophilic inflammation. In 3-month-old puppies, the zones of precornification of the internal root sheath and hair shaft of hair follicles appeared elongated; layers were mildly distorted and the changes continued into the zones of early cornification.

### Electron Microscopy

**Figure 4:**
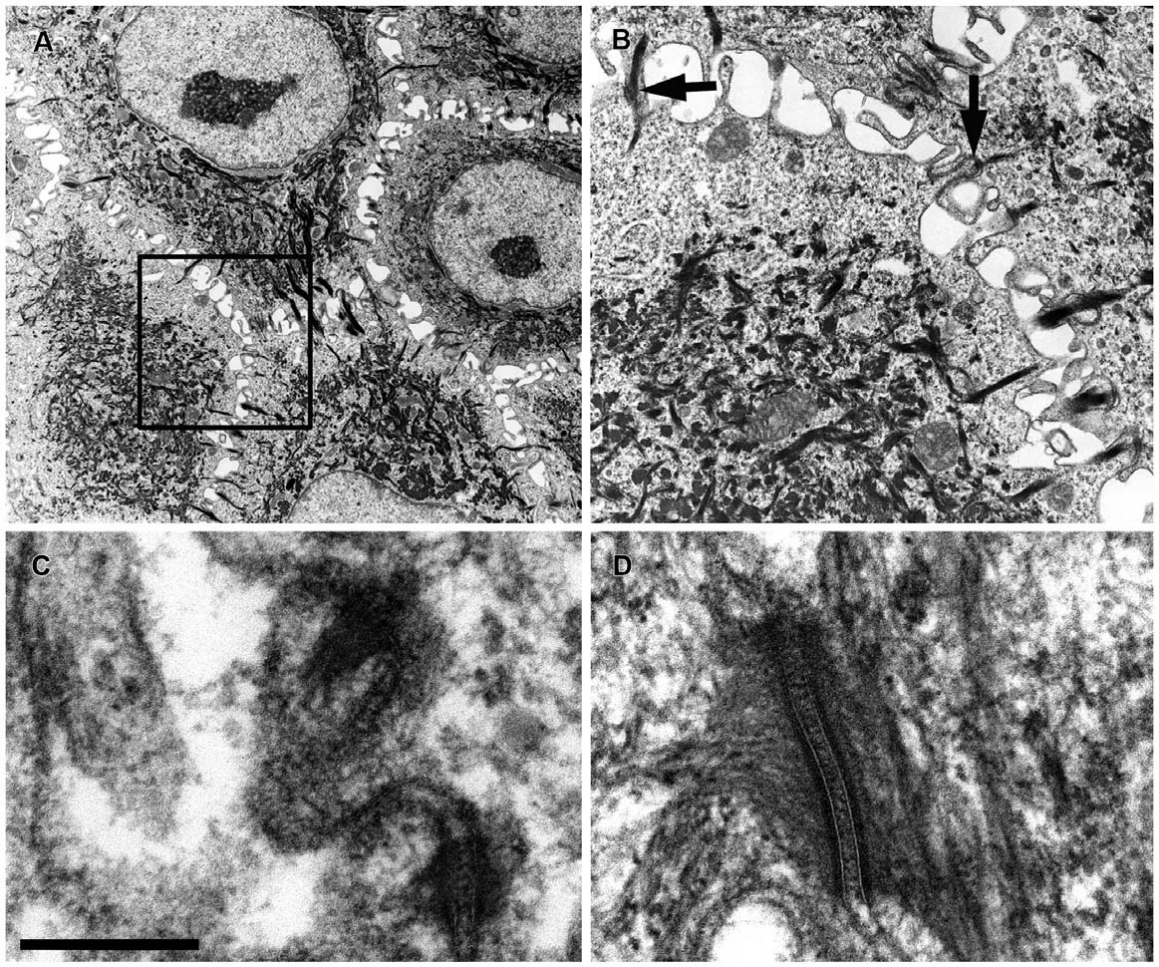
Ultrastructural findings for 3-month-old puppies with ectodermal dysplasia-skin fragility syndrome and PKP1 deficiency. Panel A: Transmission electron microscopy of carpal pad epidermis reveals widening of intercellular spaces between keratinocytes and retraction of keratin filaments from the cell membrane. Panel B: Higher magnification photomicrograph (Box insert from Panel A) demonstrates reduced numbers of desmosomes attaching keratinocytes as well as the presence of small, partially formed desmosomes (arrows). Panels C and D: A higher magnification confirms that desmosomes appear small and rudimentary in an affected dog (C) compared to those of a normal dog (D). Bar = 1 µm.

Examination of lesional epidermis by TEM revealed widened intercellular spaces but retention of convoluted, interdigitating plasma membranes (Figure 4A,B). In several affected areas, desmosomes appeared greatly reduced in number, in association with a reduced tonofilament density in the peripheral cytoplasm of keratinocytes. Very small, partially formed desmosomes lacked distinct inner and outer dense plaques and connected to thin tonofilament bundles (Figure 4C). A layer of densely packed tonofilaments encircled the nucleus in non-overtly acantholytic keratinocytes that had reduced desmosomes and a paucity of tonofilaments in the peripheral cytoplasm, consistent with collapse of the intermediate filament cytoskeletal network. Tonofilaments formed a single, large, dense rounded aggregate in the cytoplasm of some keratinocytes that appeared to displace the nucleus. In all, desmosomes from affected dogs (Figure 4C) had a profound alteration of their ultrastructure compared with those of normal dogs (Figure 4D).

### Protein Immunomapping

**Figure 5:**
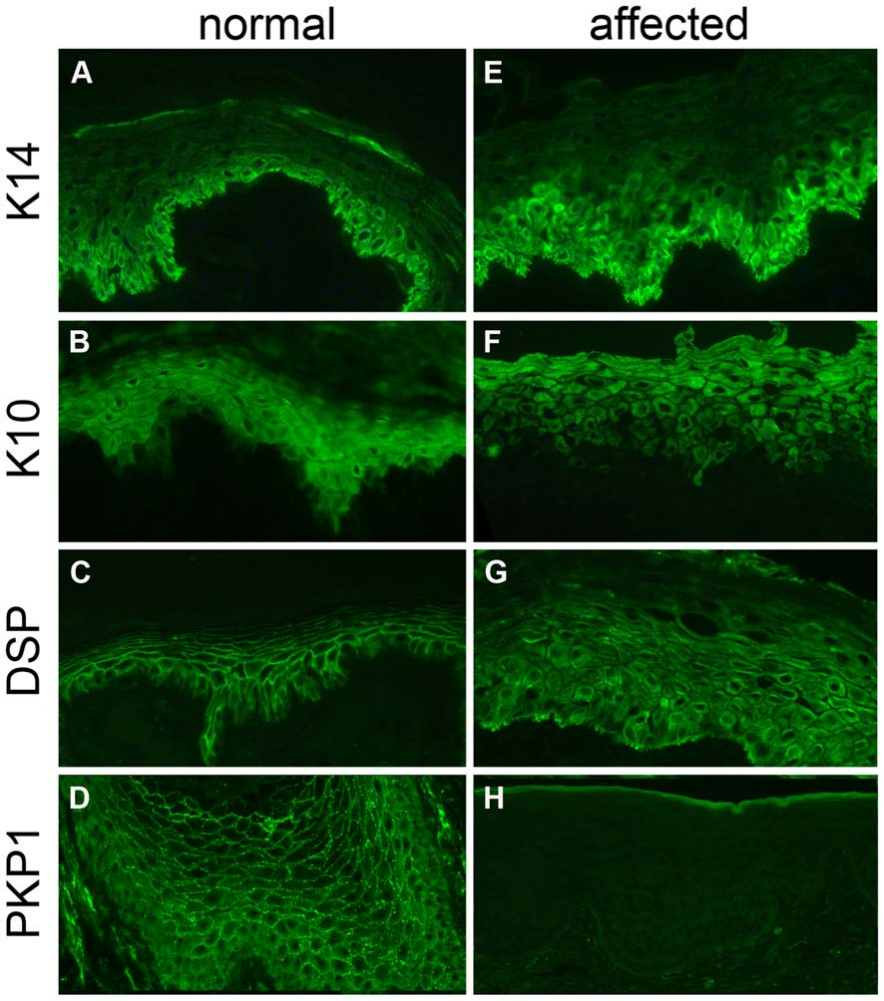
Expression of selected keratin and desmosomes proteins in normal and affected Chesapeake Bay retriever dogs. Panels A–D: normal age- and breed-matched control dog footpad E–H: affected dog footpad. Panels A, E: keratin K14 was expressed in the cytoplasm of basal keratinocytes of both affected and normal dogs, but the staining pattern was more heterogeneous in affected dogs; some mid-epidermal keratinocytes expressed K14 in affected but not normal dogs. Panels B, F: in normal dogs, keratin K10 was seen with an homogeneous staining in suprabasal keratinocytes (B); in affected dogs, the staining intensity was heterogeneous within and between keratinocytes. Panels C, G: desmoplakin (DSP) staining revealed a typical fishnet intercellular pattern in normal dog skin (C), while it was cytoplasmic and heterogeneous in affected dogs (G). Panels D, H: Using the monoclonal antibody specific for armadillo-repeats of plakophilin-1 (PKP1), this molecule was detected intercellularly, with a dotted pattern, in the superficial epidermis of the normal control CBR (D); this pattern was not seen in samples from affected dogs (H). All panels were shot at ×20 magnification.

Indirect immunofluorescence (IF) performed with a panel of antibodies specific for selected epidermal keratins and desmosomal proteins revealed notable differences in staining patterns between samples of normal and affected CBR dogs (Figure 5). In the latter, however, all biopsies from grossly affected or more normal appearing ear and footpad exhibited similar changes. Staining for keratins K14 (Figure 5A,E) and K10 (Figure 5B,F) was homogeneous in normal dogs, but it was heterogeneous in intensity in affected puppies. Moreover, the expression of K14 was not restricted to basal or juxta-basal keratinocytes in affected dogs (Figure 5F). Desmoplakin was visible with an intercellular pattern in normal dogs, but it was cytoplasmic and heterogeneous in affected puppies (Figure 5C,G). Staining for PKP1 revealed the presence of the protein in the differentiated epidermis of normal CBRs with a typical intercellular and often dotted pattern; in contrast intercellular PKP1 was not seen in the epidermis of affected animals. This lack of immunoreactivity for PKP1 was seen both with the polyclonal antiserum specific for the aminoterminus of this protein (not shown) and with a monoclonal antibody targeting the armadillo repeats of PKP1 (Figure 5D, H). Differences in staining patterns between normal and affected samples were not seen for desmoglein-1, desmoglein-3, desmocollin-1, E-cadherin and plakoglobin (not shown).

Overall, the immunomapping profile of affected dogs was consistent with lesion formation due to an absence of PKP1 leading to a detachment of desmoplakin from the desmosomal plaque and its retraction to the cell cytoplasm. As a likely consequence of these changes, keratin filaments condensated and aggregated around the nucleus.

### Mutational Analysis and Genotyping

**Figure 6:**
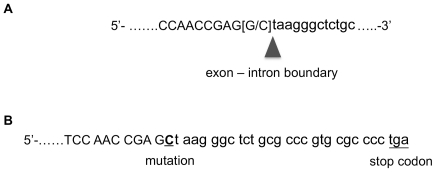
Mutation in *PKP1* causing ED-SFS in Chesapeake Bay retrievers. Panel A: Transition from G-to-C at intronic splice donor site at the beginning of the first intron ([G/C]). Panel B. Consequence of the mutation described in panel A. The intronic splice donor site is destroyed by the transition from a G to a C resulting in a continual read through to a premature stop codon. Nucleotides in capitals are from exon 1 and those in small letters from intron 1.

All exons of *PKP1* including the exon-intron boundaries were sequenced and the sequence data compared to those in GenBank (NCBI). The product sizes are listed in Table S1. Nucleotide sequencing revealed a G-to-C conversion at the IVS1 splice donor site of the first intron (Figure 6A), which was homozygous in affected puppies and heterozygous in their parents available for testing. This conversion results in a destruction of the intronic splice donor site resulting in a continual read through to a premature stop codon located nine codons downstream from the mutation (Figure 6B). As a consequence, the mutated protein is predicted to be truncated and composed of 75 instead of 749 aminoacids.

**Figure 7:**
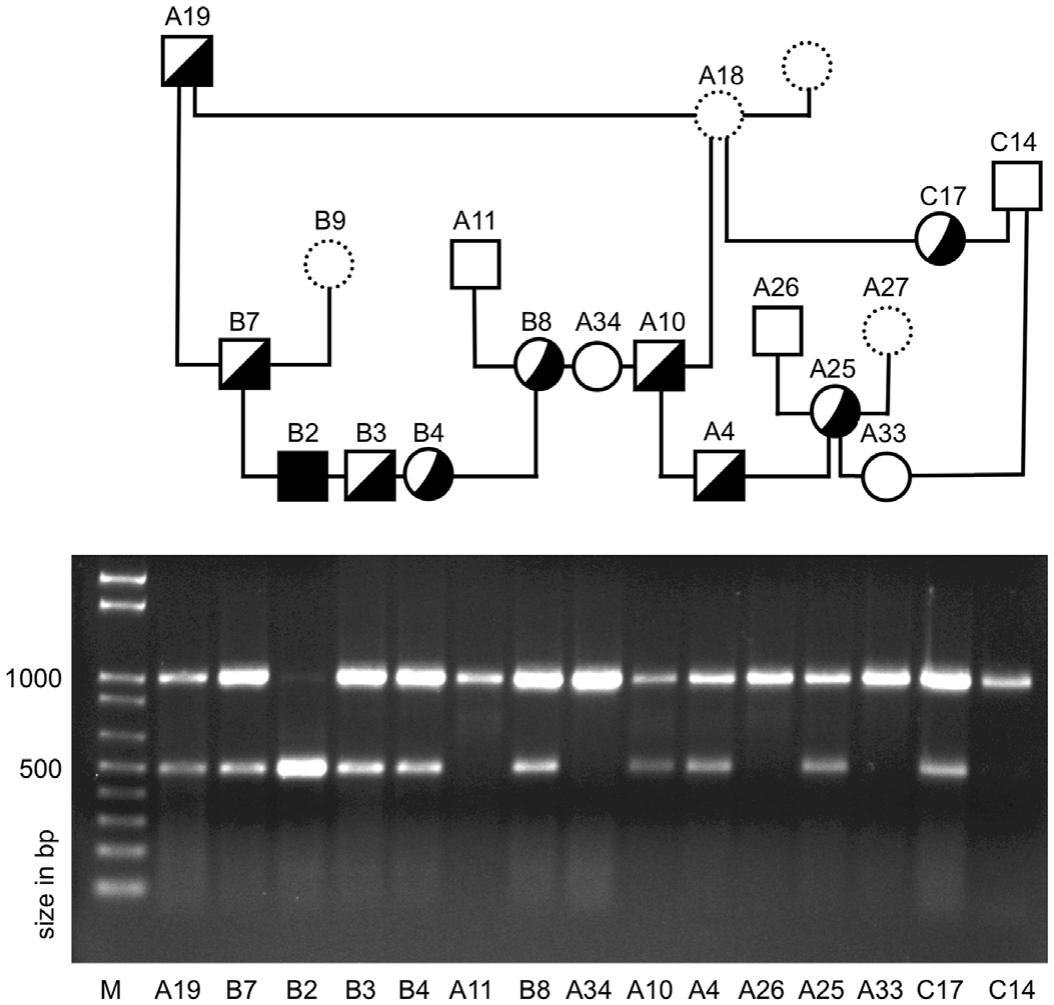
Detection of the G-to-C mutation in a family of purebred Chesapeake Bay retrievers. DNA was isolated from tissues or whole blood, amplified by PCR, digested with a restriction enzyme that cut if the mutation was present, and visualized on an agarose gel. Symbols indicate the genotype as determined by the DNA results, which are shown beneath the pedigrees. Squares = males, circles = females; open symbols = homozygous normal; filled in symbols = affected with ED-SFS and homozygous for the mutation; half-filled symbols = heterozygous for the mutation; dotted-line symbols = no DNA or clinical information available. Approximate sizes (in bp) of the DNA fragments are indicated by comparison to the marker on the left of the panel.

Furthermore, the G-to-C mutation created a restriction enzyme cutting site within the PCR product used to sequence exon 1, which resulted in two fragments of 493 and 501 bp in length. These restriction fragment length polymorphisms were then used to genotype samples from 19 CBRs: three puppies from two litters affected with ED-SFS, each of their four clinically normal parents, and ten related clinically normal CBRs. One unrelated normal dog (American bulldog) was also tested as a normal control. A partial pedigree with the RFLP results is shown in Figure 7. As expected, all of the affected puppies tested homozygous for the mutation (C/C), the parents heterozygous (G/C) and the unrelated control homozygous for the normal allele (G/G). Only three littermates to the three affected puppies were available at the time of testing, and all three were heterozygous for the mutation. The common ancestor and four close relatives were also heterozygous for this mutation. Five relatives were homozygous normal.

## Discussion

In this paper, we report a novel spontaneously-arising congenital and hereditary disease of dogs caused by a mutation in *PKP1*, which results in a very short protein, abnormal and dysfunctional superficial epidermal desmosomes, profound keratinocyte acantholysis and superficial epidermal sloughing occurring at birth. These changes are characteristic of ED-SFS with PKP1 deficiency, a very rare genetic skin disease of humans. The availability of an inbred line of dogs harbouring such spontaneous mutation could be useful for the generation of PKP1-null puppies in whom, at least theoretically, gene therapy could be attempted.

Puppies with ED-SFS exhibit most of the clinical signs seen in children with this syndrome (reviewed in [11]). In both species, the disease most often occurs at birth. Affected dogs are first noticed to have an unusual pale and fragile skin when first rubbed dry after delivery; in contrast some children with ED-SFS exhibit erythroderma at birth [2]. When diseased dogs and children age, the main anomalies are skin fragility and fissuring at sites of friction and around mucosae. Palms and soles – and their equivalent, dog footpads – exhibit a unique combination of hyperkeratosis and erosions. The coexistence of skin fragility, fissuring, hypotrichosis and abnormal hair is an unusual combination of signs among EB variants; when present this constellation of lesions altogether enhances the suspicion of ED-SFS. The growth delay and hair abnormalities reported in children were also observed in the three puppies kept alive until three months of age.

In children and puppies with this disease, light microscopy first reveals a widening of intercellular space with dissociation between epidermal cells in the superficial epidermal layers leading to epithelial sloughing (i.e. erosions). Acantholysis is associated with the aggregation of the keratin cytoskeleton (dyskeratosis). As dogs age, the epidermis becomes hyperplastic and hyperkeratotic while acantholysis decreases. Transmission electron microscopy is useful to demonstrate a reduced number of hypoplastic desmosomes with separation of keratin intermediate filaments from desmosomal plaques. These ultrastructural anomalies are shared with acantholytic suprabasal EB with desmoplakin deficiency [18]. In humans with *PKP1* mutations, the reduction of desmosomal plaque size and desmosome number in lower suprabasal keratinocytes mirrors the genotype of the patients: reductions are highest in *PKP1*-null homozygotes than in heterozygotes [19].

In children and puppies with ED-SFS, immunostaining for desmosomal proteins usually reveals a marked reduction to a complete absence of PKP1 although some carboxyterminal mutations might lead to residual PKP1 immunostaining in some children with ED-SFS [11]. Immunostains also are useful to demonstrate the detachment of desmoplakin from juxtamembrane areas and its migration to the cytoplasm along with condensation of the keratin cytoskeleton, these observations providing an immediate insight on the cellular mechanism of lesion formation.

While a variety of mutations in *PKP1* have been described in humans, an unusually large proportion of them lie in splice donor and acceptor sites such as the IVS1. [8], [11]. In general, only about 10% of genetic diseases described in humans are caused by splice site mutations [20]. Thus, it is interesting that the putative mutation found in the dogs described here occurs also at a splice site. The consequences of splice site mutations are somewhat harder to predict as cryptic donor and acceptor sites may be used for transcription that may lead to exon skipping or run on reads [20]. If the base change found in the CBRs with ED-SFS had been found in humans with the same disease, there would have been a good chance that a cryptic splice donor site would have been used only several base pairs into the first intron of human *PKP1* (www.mutationtaster.org). However, because of a sequence difference between human and canine *PKP1*, a premature stop codon was encountered before an alternate splice donor site could be used in our dogs. In theory, the premature stop codon is predicted to lead to a truncated protein (67 amino acids plus 8 from the translation of intron 1 instead of the predicted 749 amino acid long canine PKP1). Importantly, this mutation is expected to result in a loss of most of the head and all nine armadillo-domains that are critical to the binding of PKP1 to its partners desmoglein-1, desmocollin-1 and desmoplakin, thereby rendering PKP1 completely dysfunctional [21]. Unfortunately, RNA could not be preserved at the time, and, thus, it was not possible to assess the direct consequences of the mutation. In support of the mutation prediction, however, the anti-PKP1 antiserum used for the immunostaining of PKP1 in the CBRs with ED-SDS showed no binding, even though it was directed against the amino terminus of the protein. It is possible that the antiserum did not have a large enough epitope left in the PKP1 head for its binding, but it is also conceivable that no protein was produced. This likely absence of a functional PKP1 is supported by the lack of identification of this protein in affected dogs using a monoclonal antibody directed against carboxy-terminal armadillo repeats of PKP1. Furthermore, in cases where the mRNA and the resulting protein are so severely truncated, nonsense-mediated mRNA decay prevents production of the protein as a quality control/surveillance measure [22]. If a short remnant of PKP1 were still to be translated, it could also theoretically lead to a dominant-negative recruitment of desmoplakin and plakoglobin to the cytoplasmic carboxyterminal tail of desmosomal cadherins, thereby interfering with normal desmosome formation and structure.

In summary, we reported herein a novel canine skin disease that mirrors clinical, microscopic, ultrastructural and *PKP1* mutation characteristics of ED-SFS of humans. With the selective mating of mutation-carrying CBRs and the generation of *PKP1*-null puppies, one could envision the possibility of evaluating the feasibility of gene therapy for this rare disease of children, as such intervention cannot be translated directly from the laboratory to human individuals. The main limitation of this spontaneous animal model, however, is the requirement of purpose-mating rare carrier dogs to obtain affected puppies that, in the absence of treatment effect, would have to be euthanized promptly for humane purposes. Alternatively, with a genetic test now available, ED-SFS could be rapidly eliminated from this breed with the testing of all at risk CBRs and the subsequent removal of mutation carriers.

## Supporting Information

Table S1 **PCR primers used for amplification and sequencing of the dog** ***PKP1*** **gene.**
(DOC)
