# Supplementary material for: Deficient Plakophilin-1 Expression Due to a Mutation in PKP1 Causes Ectodermal Dysplasia-Skin Fragility Syndrome in Chesapeake Bay Retriever Dogs
Source: PLoS One. 2012 Feb 22;7(2):e32072. doi: 10.1371/journal.pone.0032072 (PMC3284538; doi:10.1371/journal.pone.0032072)
Supplement: Table S1 — PCR primers used for amplification and sequencing of the dog PKP1 gene. (DOC) [file pone.0032072.s001.doc]

| **Exon** | **Primer** | | | | **PCR Temp. ^0^C** | **Product (bp)** |
| --- | --- | --- | --- | --- | --- | --- |
|  | **Name** | **Sequence (5’-3’)** | **Length (bp)** | **Tm ^0^C**  **(IDT)** |  |  |
| 1 | PKP1Exon1F | CTTGCTCCTTCCTGCTCAAG | 20 | 56 | 68 | 994 |
|  | PKP1Exon1R | AGCGCATACTTTGTCCCTGA | 20 | 57 |  |  |
| 2 | #7_PKP1.F1 | GCAATATCCAATCAGCCCGAACAG | 24 | 63 | 62 | 1227 |
|  | #7_PKP1.R1 | AGGGGCCTGGTATGAGCAAAGTCG | 24 | 58 |  |  |
| 3 | PKP1E3F | TCACTTCATGGACCAAAGCA | 20 | 54 | 60 | 392 |
|  | PKP1E3R | CGAGCGGATGATGTATTTCC | 20 | 53 |  |  |
| 4 | PKP1E4F2 | TTCACGGAGAGTGTCCCAAG | 20 | 57 | 62 | 762 |
|  | PKP1E4R2 | TGCCTTCCACCACAAACATC | 20 | 56 |  |  |
| 5-6 | #5_PKP1.F1 | AGGCGCTTGAGACCCCGTTAGT | 22 | 63 | 62 | 1882 |
|  | #5_PKP1.R1 | TTTCCCAGCATCCGACATAGACAT | 24 | 58 |  |  |
| 7 | #4_PKP1.F1 | CCCTCTTCGTGCTTTGCTTATTAC | 24 | 56 | 60 | 2108 |
|  | #4_PKP1.R1 | TGAGGGGGCCAGATGTAGTA | 20 | 57 |  |  |
| 8a | PKP1Exon8aF | CTGGGGAGGAGAAGAGGAAG | 20 | 56 | 60 | 496 |
|  | PKP1Exon8aR | CTGTGGCTCAGCCTCCAT | 18 | 57 |  |  |
| 8 | PKP1Exon8F2 | CTGTGTGCAGGACGGAAGG | 19 | 58 | 59 | 660 |
|  | PKP1Exon8R2 | CTTCCAGGCGCTTCTGATCT | 20 | 57 |  |  |
| 9 | PKP1Exon9F | GCCTCTGTGCGTGTCTGTAG | 20 | 58 | 62 | 543 |
|  | PKP1Exon9R | ACTGCGGTATGAGACCCAAA | 20 | 56 |  |  |
| 10-11 | PKP1E10E11F2 | GGACCTTCTCTGCGTCCAAG | 20 | 58 | 61 | 992 |
|  | PKP1E10E11R2 | GTCTGTCTTTGCCCGTGCTT | 20 | 58 |  |  |
| 12 | #2_PKP1.F1 | GCCCCTCGCACACTTCCTTGATTC | 24 | 62 | 60 | 571 |
|  | #2_PKP1.R2 | ACGGCCCCCGAGACAATCCTAAG | 24 | 63 |  |  |
| 13 | #1_PKP1.F1 | TCCAGAGCCACCGAGTCACAAC | 22 | 61 | 62 | 810 |
|  | #1_PKP1.R1 | GGAGGCCCAGACGCACTTCACTA | 23 | 63 |  |  |
